# Network Control Models With Personalized Genomics Data for Understanding Tumor Heterogeneity in Cancer

**DOI:** 10.3389/fonc.2022.891676

**Published:** 2022-05-31

**Authors:** Jipeng Yan, Zhuo Hu, Zong-Wei Li, Shiren Sun, Wei-Feng Guo

**Affiliations:** ^1^ Department of Nephrology, Xijing Hospital, The Fourth Military Medical University, Xi’an, China; ^2^ School of Electrical Engineering, Zhengzhou University, Zhengzhou, China; ^3^ State Key Laboratory of Oncology in South China, Collaborative Innovation Center for Cancer Medicine, Sun Yat-sen University Cancer Center, Guangzhou, China

**Keywords:** personalized omics, network control principles, tumor heterogeneity, precision medicine, cancer individual patients

## Abstract

Due to rapid development of high-throughput sequencing and biotechnology, it has brought new opportunities and challenges in developing efficient computational methods for exploring personalized genomics data of cancer patients. Because of the high-dimension and small sample size characteristics of these personalized genomics data, it is difficult for excavating effective information by using traditional statistical methods. In the past few years, network control methods have been proposed to solve networked system with high-dimension and small sample size. Researchers have made progress in the design and optimization of network control principles. However, there are few studies comprehensively surveying network control methods to analyze the biomolecular network data of individual patients. To address this problem, here we comprehensively surveyed complex network control methods on personalized omics data for understanding tumor heterogeneity in precision medicine of individual patients with cancer.

## Introduction

Increasing studies on cancer genomics data have revealed that individual heterogeneity of cancer patients is one of the main reasons for no substantive breakthrough in cancer treatment methods. With the recent development in high-throughput omics technology, data resources have become available for cancer research, such as genomic and transcriptomic data (1, 2). Personalized omics data of individual patients should be analyzed for understanding the tumor heterogeneity of cancer diseases. The key challenges are how to integrate multi-level omics data, such as genomic and transcriptomic data, for understanding the regulatory mechanism in individual patients, and how to identify cancer-related drug targets (3). Therefore, it is of great theoretical significance and clinical application value for designing computational methods through integration of the omics data of individual patients and screening for drug targets related to phenotype transitions of these patients.

**Figure 1:**
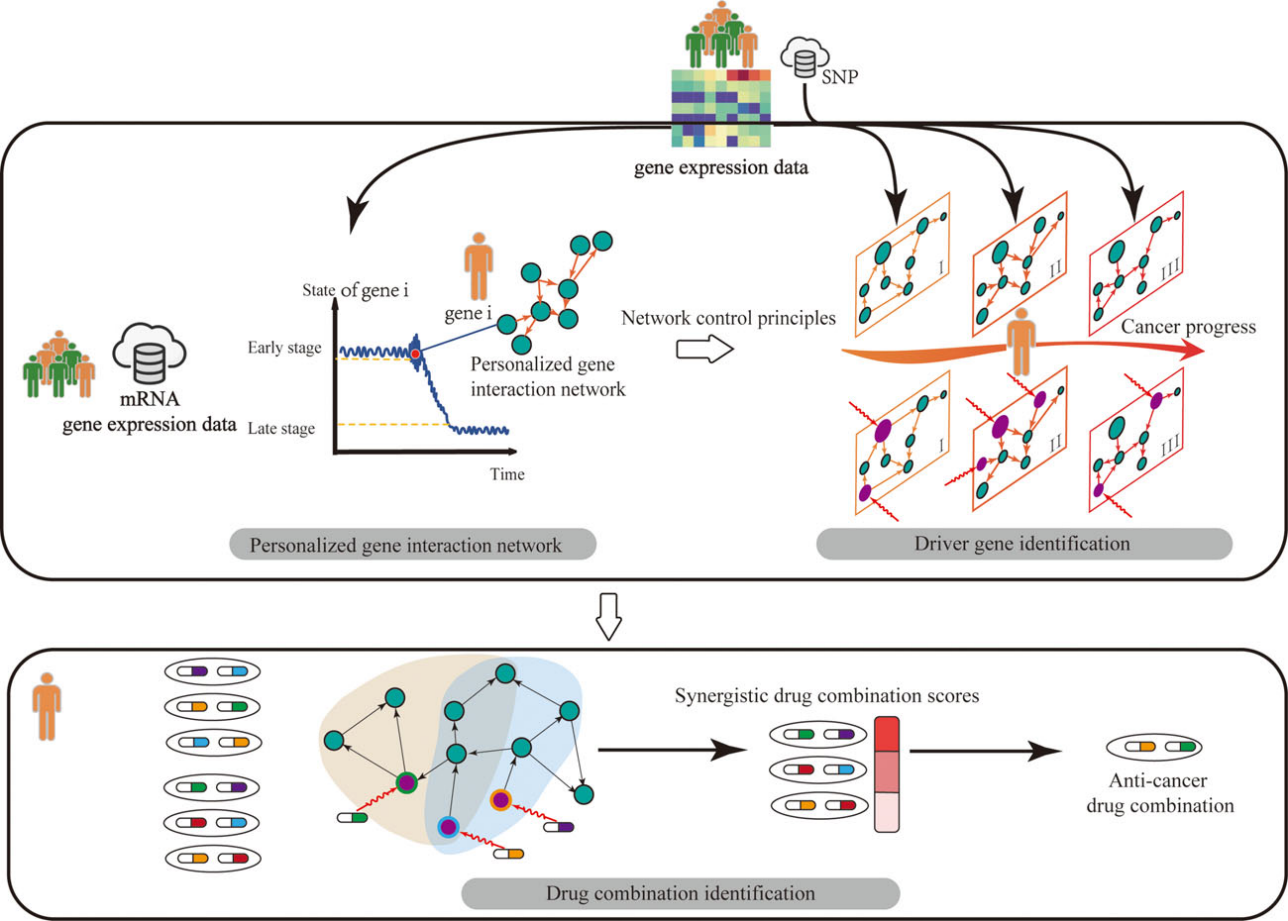
Overview of our review. The contents of our review consist of three parts. Firstly, we summarized the works to construct personalized gene interaction network from genomics of individual patients. Then on the personalized gene interaction networks, we pointed out how to identify personalized driver gene by using network control tools. Finally, we described how to discover synergistic drug combinations by targeting personalized driver genes.

Modern medical studies have shown that cancer is generally an outcome of the dysfunction of related dynamic systems. From the system biology perspective, cancer can be driven by the state transition of key driver genes, which can lead to the dysfunction of molecular networks (e.g., gene regulation networks or signal transduction networks) that regulate molecular pathways and cellular processes. Moreover, the state of biomolecules (e.g., gene expression value) with complex dynamic characteristics in individual patients changes with time and environmental conditions (**Figure 1**). To understand the dynamics of individual patients, the regulatory mechanism of molecular networks needs to be understood from the perspective of network control theory. This theory considers the state variables of a high-dimensional dynamic system as a complex network and studies how to effectively control the state of driving variables through control signals in the system with optimal control objectives (such as minimum number of controllers or minimum energy), thus changing the network state to the desired stable state (4).

Although traditional control theory (5) has been studied extensively, it is not suitable for a biological network system with numerous nodes (e.g., genes). Network control methods provide the technology for analyzing biomolecule networks with complex dynamic characteristics and quantifying their ability to intervene the biomolecule system of individual patients through proper control signals (6, 7). Moreover, researchers have made progress about network control principles. However, few studies comprehensively surveyed network control methods to analyze the biomolecular network data of individual patients. Considering these facts, this study provided a comprehensive survey for complex network control methods on the multi-omics data of individual patients including methods for personalized gene interaction network construction, network control principles, driver gene prediction, and drug combination identification (**Figure 1**), which aims to reveal the molecular mechanism and regulation law of personalized biomolecular systems for the diagnosis, prevention, and treatment of individual patients.

## Datasets

With the development of cancer genomics technology, many data resources are available for understanding the cancer mechanism. In the past decade, a large amount of cancer genome data from large-scale cancer genomics projects facilitated the development of computational methods for mining personalized omics data of individual patients and understanding tumor heterogeneity in cancer precision medicine. Among these cancer genomics projects, The Cancer Genome Atlas, an important database for mining cancer omics data (2), has created a genomic panorama of different cancers. It currently contains 33 cancer types and more than 20,000 samples. The Cancer Cell Line Encyclopedia is a compilation of gene expression, chromosomal copy number, and massively parallel sequencing data from 1457 cell lines. It provides the pharmacological activities of 24 anticancer drugs in 504 cell lines (8, 9). Gene Expression Omnibus (GEO) is a public repository of functional genomics data currently storing approximately 23,002 public series submitted directly by 168,607 laboratories. This series comprises 4,851,647 samples derived from more than 1600 organisms (10).

BioGPS (11) is an online gene annotation database integrating 150 resources. It can query gene name information, chromosome location, gene function, transcript information, encoded protein information, and related protein names. However, this database cannot provide a detailed gene annotation list, and therefore, users find it difficult to annotate the genes for a large number of samples. The cancer gene census (CGC) data (12) offers a detailed list of driver genes that have been experimentally verified as cancer driver genes (13–15). The Network of Cancer Genes (NCG) (16) is a database that collects and annotates cancer genes from a large amount of cancer sequencing data. This database contains 2372 genes, including experimentally verified cancer driver genes.

## Construction of Personalized Gene Interaction Network

A biological system is a complex dynamic multi-scale system involving different time, space, and functions. Cells contain genes that store information, proteins, and metabolites and perform biological functions for forming basic functional modules. A complex biological system is composed of multiple functional modules. In system biology, the key to constitute a biological system is not its components (e.g., genes, proteins, and small biological molecules), but their interactions with components having different properties. These interactions constitute the regulatory network controlling different biological functions.

The rich information can be obtained from the high-dimensional data of samples of individual patients. However, the individual genomics data of these patients are often limited and incomplete. Therefore, methods to ensure complete use of personalized genomics data for designing effective gene interaction network construction algorithms of individual patients must be developed. The personalized gene interaction network represents which gene pairs are involved in the disease development for each patient. Because the principles of the personalized network dynamics are hidden, it is important to reconstruct the personalized state transition networks with the personalized genetic data (e.g., expression profiles). It is a key challenge to unravel the dynamic nature of gene regulation during a biological process in systems biology.

Current gene interaction network construction methods, such as Gene Network Reconstruction tool (GNR) (17), dynamic cascaded method (DCM) (18), and Hotnet2 (19), use gene expression data of population cancer patients. Although these gene regulation networks can reflect the gene interaction mechanism of the disease, they cannot describe the gene interaction relationship of individual patients. Numerous single-sample gene interaction network construction methods have recently been proposed. Several common techniques including Single Sample Network construction method (SSN) (20), Paired Single Sample Network construction method (Paired-SSN) (21), Single Pearson Correlation Coefficient calculation method (SPCC) (22, 23), and Cell Specific Network construction method (CSN) (24), and Linear Interpolation to Obtain Network Estimates for Single Samples (LIONESS) (25) were introduced as follows. In **Table S1 of Supplementary Tables**, we gave a summary of these methods including brief descriptions and input data for constructing personalized gene interaction network.

1) SSN
SSN is a statistical method to construct an individual-specific network based on statistical perturbation analysis of a single sample against a group of given control samples (20). For the SSN method, the co-expression network of the tumor sample network or normal sample network for each patient is constructed based on statistical perturbation analysis of one sample against a group of given reference samples (e.g., choosing the normal sample data of all of the patients as the reference data).
2) Paired-SSN
For the paired-SSN method (21), the co-expression network of the tumor sample network and normal sample network for each patient is firstly constructed in the same way as for the SSN method. Then, the personalized differential co-expression network between the normal sample network and tumor sample network can be constructed in which the edge will exist if the *P*-value of the gene pair is less than (greater than) 0.05 in the tumor network but greater than (less than) 0.05 in the normal network for their corresponding patient.
3) SPCC method
To overcome the difficulty in obtaining correlations or edges from one sample, the SPCC approach (22, 23) was developed by decomposing each PCC measurement into multiple additive elements that form a new vector embedding correlation-like information of two variables for one sample.
4) LIONESS
LIONESS does not rely upon differential analysis between the tumor sample and a group of normal samples, and it reconstructs the individual specific network in a population of tumor samples as the personalized gene state transition network for each tumor sample (25). LIONESS constructs the state transition network by calculating the edge statistical significance between all the tumor samples and the tumor samples without a given single sample.
5) CSN
The CSN method is derived from a theoretical model based on statistical dependency (24), which can be viewed as data transformation from the “unstable” gene expression data to the “stable” gene association data. CSN designs a statistic for gene pairs and can obtain the P-value corresponding to the edge between genes by the statistic.

We should note that conditional or partial sample-specific correlation network can be generally used to eliminate the indirect co-expressions between genes (26, 27). Furthermore, the reference reliable gene/protein interaction network are generally used to take overlapped edges from the original gene co-expression edges, forming the final personalized gene interaction networks for the above methods. However, the current single-sample gene regulation network construction methods ignore temporal data of individual patients (28), and their accuracy and stability need improvement.

## Network Control Principles

A core concept in network science is to control and intervene on network dynamics (4). Network control methods have recently received extensive attention (29–36). Therefore, network control methods are better than the traditional control concept in revealing the dynamic characteristics of biological networks with a lot of noise in edge weight. In particular, considering the gene expression profiles in normal and tumor samples as the respective state of a given patient, network control tools aim to detect a small number of driver nodes by the input signals related with the state transition of individual patient depending on adequate knowledge of the network structure. The input signals may be oncogene activation signals such as gene mutation or metabolites changes in specific tissue. The “controllers” in network control problem for molecular networks mean the genetic or environment factors which produce the oncogene activation signals. As per our best understanding, current methods can be classified as directed and undirected network control methods.

i) For the control methods of directed networks, Liu et al. (37) studied the structural control of directed networks and proposed Maximum Matching Sets-based controllability methods by referring to the structural controllability theory of linear systems, which has greatly inspired the promotion of research of network control methods and applications. Although these network control tools have been applied to biomolecular systems, some interesting properties of biological systems have also been discovered. For example, driving mutant genes (38) and drug targets (39) were found in cancer datasets, and driving metabolites were detected in human liver metabolic networks (40). However, these tools only describe the linear dynamic behavior of the network and are not sufficient for completely characterizing the complex nonlinear dynamic system. Recently, a Feedback Vertex Set (FVS)-based control method based on the framework of feedback vertex set control theory (FC) that can be used to study network systems with nonlinear dynamics was proposed. However, for the FVS-based control method, not only the network structure needs to be known but also the functional form of the governing equation must satisfy certain properties (41, 42). Zanudo et al. applied FVS-based control to directed networks (43). By comparing FVS predictions with those of MMS-based controllability methods, they identified topological features underlying different observed phenomena.
ii) For the control methods of undirected networks, Yuan et al. proposed an accurate control method (44) that can identify the minimum set of driver nodes in the undirected networks. Because the precise control method only describes the linear dynamic behavior of biological networks, it cannot be used to accurately describe their nonlinear dynamic behavior. To overcome the aforementioned problems, some researchers proposed minimum dominating set (MDS)-based control methods (45). These methods, however, have a strong assumption on control signals, that is, these signals can independently control their neighborhood nodes. However, most controllers cannot satisfy the strong conditions; therefore, FVS-based methods of undirected networks (namely NCUA) based on the framework of feedback vertex set control theory (FC) have been proposed (46). Since most current methods are designed based on the time-invariant network system, temporal networks can accurately describe the characteristics of cancer omics data. Thus, more accurate network control methods need to be further developed to accurately understand the dynamic characteristics of the network.

**Figure 2:**
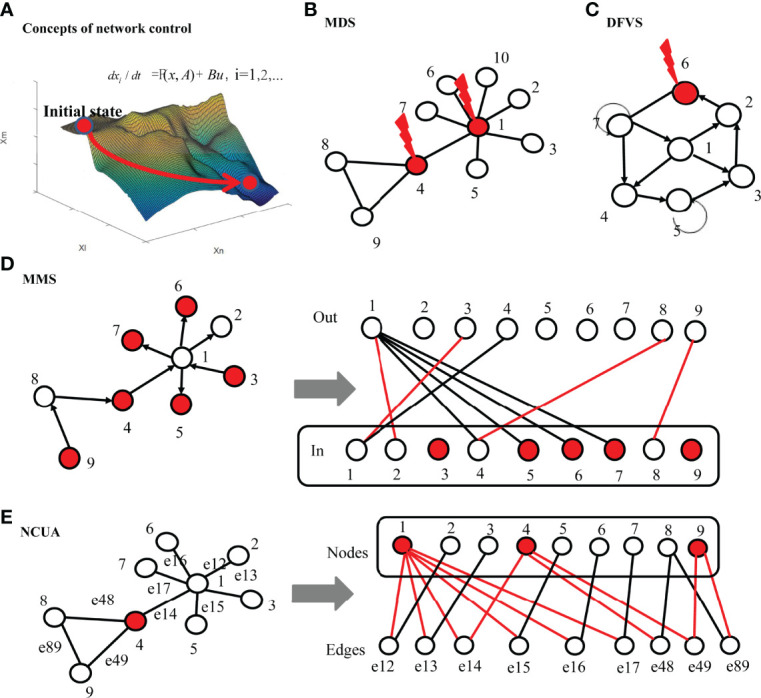
The principles of different network control methods. **(A)** Concept demonstration of network control methods. Network control tools aim to detect a small number of driver nodes which form the input matrix and are injected by the input signals for driving the state transition of high dimension networked system depending on adequate knowledge of the network structure. **(B)** MDS based control methods. If the connected edges of MDS are removed, there will be no edges in the network. By assuming that the driving node can independently control all neighbor nodes, the minimum dominating set (MDS) in the undirected network is taken as the set of driver nodes, and the red node represents the minimum driving node. **(C)** DFVS based control methods. The red nodes represent the minimum set of feedback nodes (FVS), that is, if the connected edges of FVS are removed, there will be no loops in the network. For FVS based control methods, by controlling the nodes in FVS, the whole system can be transformed from one stable attractor to another attractor. **(D)** MMS based control methods. The directed network is transformed into a bipartite graph. For the bipartite graph, the upper side represents the out degree of the original network, while the bottom side represent the in degree of the original network nodes. If there is an edge from one node to another node in the original network, an edge connecting these two nodes is added to the bipartite graph. According to the maximum matching of bipartite graph, the maximum matching (i.e., red edges) can be obtained, and 6 unmatched nodes (i.e., red nodes) can be found in the bottom side of bipartite graph. By controlling these 6 nodes, the system structure can be completely controllable for MMS based control methods. **(E)** NCUA based control methods. Firstly, the original undirected network is transformed into a bipartite graph, in which the upper side represents the nodes of the original network and the bottom side represent the edges of the original network respectively. Then, the nodes covering the nodes on the bottom side (i.e., red nodes) are obtained in the bipartite graph and are considered as driver nodes for the NCUA method. The red edges represent the links between the driver nodes and the nodes of bottom side in the bipartite graph.

To easier understand these network control methods, we gave the concept comparisons including the network types and targeted states and input and network dynamics between different network control method including MMS, MDS and DFVS and NCUA (**Table S2 of Supplementary Tables**). In **Figure 2**, we intuitively explained these methods. We summarized some key points of different structure network control methods as follow:

i) The MMS control methods investigate the controllability of directed structural networks with linear or local nonlinear dynamics through a minimum set of input nodes and they only give an incomplete view of the network control properties for a system with nonlinear dynamics.
ii) MDS control method studies the controllability of undirected networks by assuming that each driver node in the MDS model can control its associated edges independently in the undirected networks. Since MDS works with the strong assumption that the controllers can control its outgoing links independently, it requires higher costs in many kinds of networks which may underestimate the structural control analysis of undirected networks;
iii) NCUA and DFVS study the structural network control of undirected and direct networks respectively based on the framework of FC (42). Therefore NCUA and DFVS methods ultimately depict the structure-based network control of the large-scale system with nonlinear dynamics. Since FC assumes that the functional form of the governing equations must satisfy some continuous, dissipative, and decaying properties, DFVS and NCUA may be only suitable some specialized nonlinear systems.

## Driver Gene Prediction

Using new methods, researchers have recently made some progress in predicting cancer driver genes of population cohorts. These methods are based on mutation frequency, machine learning, and complex network. (1) In gene mutation frequency-based methods, the mutation frequency of driver genes is usually assumed to be significantly higher than that of other genes (13, 47–51). However, due to the tumor heterogeneity, it is difficult to build a reliable background mutation model. In addition, these methods cannot be used to detect the low-mutated frequency and non-mutated cancer driver genes, because a part of driver genes is mutated at high frequencies (>20%), while most of cancer mutations occur at intermediate frequencies (2–20%) or even lower (52), and even many genes that play important roles in tumorigenesis are not altered on the DNA sequences, and these genes are dysregulated through various cellular mechanisms (53). (2) Machine learning-based methods (49, 51, 54–56) usually train the classifier (e.g., random forest, support vector machine) by extracting various kinds of features from different types of cancer data to predict new cancer driver genes. Although machine learning-based methods can effectively predict some cancer driver genes, because of the incomplete database, some key driver genes may be ignored, thus generating false-positive results. (3) Complex network-based methods usually assume that driver genes have obvious structural characteristics at the biological network level (19, 57, 58). Although these methods have been successfully used for detecting cancer driver genes, they are still limited to incomplete and unreliable interactions in biological network (59).

The aforementioned algorithms focus on how to identify driver genes in population cohorts but cannot be directly applied to the data of individual patients because of the following reasons. On the one hand, TCGA provides 33 cancer types with more than 10,000 samples and 3,000,000 mutation data, while the sample size for individual patients is typically very small. On the other hand, the functional characteristics of cancer mutations in population cohorts are different from those observed in individual cancer patient data with complex and unclear dynamics. Therefore, considering that network techniques such as random walk with restart (RWR) (60), network diffusion (19, 61), subnetwork enrichment analysis (62), matrix completion (63) and network structure control (6, 64–66) to predict cancer driver genes at the biological network level by incorporating the knowledge of pathways, protein-protein interactions, can deal with high-dimensional data having a small sample size, researchers proposed some network algorithms for predicting personalized driver genes of individual patients (14, 15, 67). Although these algorithms can predict personalized driver genes with important biological functions, they do not consider dynamic changes in the structure of the personalized gene interaction network, thereby leading to false-positive results and affecting the accuracy of driver gene identification. Therefore, dynamic changes in the structure of the personalized gene interaction network must be considered for inferring the evolution trajectory of driver genes and accurately understanding the cancer development mechanism.

## Drug Combination Identification

Computational methods for predicting combination drugs have recently attracted extensive attention (68). These methods can predict a large number of combination drugs with enhanced efficacy and reduced adverse effects (69), which are beneficial for providing efficient clinical treatments (70). At present, drug combination prediction methods mainly include complex network- and machine learning-based methods. (1) The complex network-based methods generally use some network optimization algorithms to predict drug combinations (71–73). However, their predictive performance relies on prior knowledge of drug targets and disease-related networks and is generally only suitable for a few specific diseases. (2) The machine learning-based methods use drug attribute information and cell line experimental data to predict combination drugs (74). The drug characteristics include chemical structure (75, 76), physical and chemical properties of the substructure and toxic modules (77), drug targets (78), and single drug dose response (79, 80). The cell line data include gene expression profile (81), transcriptome (76), pathway network (82), gene interaction network (83), microRNA expression and protein abundance (84), gene variation information, and copy number variation (78). The machine learning algorithms for drug combination prediction mainly include logistic regression (76), Bayesian network (85), random forest (86), multi-decision assemble (87), deep neural network (77), and deep residual neural network (88).

The existing methods ignore the heterogeneity of combination drugs among individual patients, and thus cannot predict effective drug combinations for individual patients. Therefore, an appropriate and effective drug combination prediction method needs to be designed by considering the information of individual patients. However, because personalized genomics data are generated from a small sample size and have a high dimension, these drug combination methods based on large samples cannot be used to accurately identify individual drug combinations from such data. Several studies have attempted to provide such recommendations through predictive models that can predict the efficacy of a drug for an input genomic profile. For instance, Sheng et al. (89) proposed an algorithm on a cell line (or a patient profile) based on similarity of the input drug and cell line to those in Genomics of Drug Sensitivity in Cancer (GDSC) and Cancer Cell Line Encyclopedia (CCLE). Recently, Drug Recommendations by Integrating Multiple Biomedical Databases (DruID) (90) utilized a Prescriptive Analytics framework based on Integer Programming on multiple public well known databases (91) for personalized drug recommendations. These methods require genomics data from cell lines and a list of genes with mutations, from a single patient, as input which ignore the personalized gene interaction for identifying personalized drug combinations. In our previous work, we used the network control theory to design a personalized drug combination prediction model, aiming to identify personalized synergistic drug combinations by targeting personalized driver genes of individual patients (6, 7). In fact, the model neglected the individual dynamic characteristics of drug activity and toxic concentration in drug combination therapy, and thus could not provide precise personalized drug combination. Therefore, a more effective personalized drug combination prediction model needs to be designed considering more information of individual patients’ omics data.

## Future Directions

Due to the complex dynamics of cancer data, some of the future directions for designing network control methods are as follows:

i)Designing Boolean network control methods. Although network control methods can analyze the dynamics of a biomolecular network, the positive and negative sign characteristics of interactions are currently ignored. For example, in a gene interaction network, the activation or inhibition regulatory relationship between genes is indicated as a connection with positive and negative signs (92). These signs of the gene interaction network become important when the network is controlled in a particular manner, whereas most current network control methods for personalized genome omics data only assume that their state of interactions are non-zero and do not make any assumptions about the sign of interactions. Therefore, designing Boolean network control methods considering the positive and negative sign characteristics of network interactions is an important future direction.
ii) Designing temporal network control methods. Driver genes influence the cell state through a combination of molecular interactions that may change dynamically during cancer progression (93, 94). At present, most network control methods are designed based on the static time-invariant network structure. However, the network structures of cancer patients differ at different cancer stages, which needs to be considered (95). Therefore, how to design reasonable temporal network control methods for inferring the evolutionary trajectory of driver genes in cancer patients needs to be determined.
iii) Predicting biomarker for individual patients based on network observability. Individual early diagnosis has become essential in precision medicine, which has thus made biomarker prediction increasingly important in drug development (96–102). Therefore, more effective methods are required to describe transitions in cancer status and to identify more biologically significant biomarkers. Network observability is dual with network controllability for network with linear dynamics (103, 104). It focuses on how to select key sensor nodes in the network to reconstruct the state of the entire network. Therefore, developing effective biomarker prediction algorithms for individual patients based on network observability is another crucial future direction.
iv) Designing network control methods on personalized single-cell data. With the development of biological sequencing technology, single-cell data provides a powerful resource for revealing the gene interaction of a single cell and understanding the tumor heterogeneity of individual patients with cancer (105). Therefore, how to design effective network control methods on the single-cell data of individual patients to predict biomarkers, driver genes, and drug targets for such patients is another important research direction.
v) Designing deep learning techniques for network controllability. Over the past decade, deep learning has become a focal topic in artificial intelligence and machine learning (106, 107).In fact, deep learning techniques have been developed for predicting the controllability robustness according to the input network-adjacency matrices (108). However, there are no related works to apply deep learning techniques especially graph based deep learning techniques (107) for studying controllability of personalized gene interaction network. Therefore, how to utilize deep learning methods to analyze controllability of personalized gene network is another interesting and important topic in the future.

## Conclusions

The genomic profiles of cancer patients are diverse and heterogeneous. These profiles are believed to be responsible for heterogeneity of drug response in cancer patients. The current main challenge in cancer precision medicine is to develop effective computational methods for finding personalized biomarkers, driver genes, and drug targets for individual patients. These personalized biomarkers, driver genes, and drug targets would help improve the outcome of cancer patients, especially those with drug resistance. As the number of samples of an individual patient is usually limited, the accuracy and reliability of statistical methods based on a large sample size (109, 110) will greatly be reduced for mining personalized omics data of individual patients. Therefore, considering the multi-omics data of individual patients, this study discusses cancer datasets, construction of gene regulation network, network structure control method, driver gene prediction, and drug combination prediction for individual patients in order to understand tumor heterogeneity in precision medicine.

## Author Contributions

W-FG developed the methodology and designed research. JY and W-FG wrote the original manuscript. JY, ZH, Z-WL, SS and W-FG revised the manuscript. SS and W-FG supervised the work, made critical revisions of the paper, and approved the submission of the manuscript. All authors contributed to the article and approved the submitted version.

## Funding

This paper was supported by the National Natural Science Foundation of China [62002329 (W-FG)], and Key scientific and technological projects of Henan Province [2102310083(W-FG)], and China postdoctoral foundation [2021M692915(W-FG)], and Henan postdoctoral foundation [202002021(W-FG)], Research start-up funds for top doctors in Zhengzhou University [32211739 (W-FG)], and open Funds of State Key Laboratory of Oncology in South China [HN2021-01 (W-FG)]. The funders had no role in study design, data collection, and analysis, decision to publish, or preparation of the manuscript.

## Conflict of Interest

The authors declare that the research was conducted in the absence of any commercial or financial relationships that could be construed as a potential conflict of interest.

## Publisher’s Note

All claims expressed in this article are solely those of the authors and do not necessarily represent those of their affiliated organizations, or those of the publisher, the editors and the reviewers. Any product that may be evaluated in this article, or claim that may be made by its manufacturer, is not guaranteed or endorsed by the publisher.
