# Supplementary material for: Network Control Models With Personalized Genomics Data for Understanding Tumor Heterogeneity in Cancer
Source: Front Oncol. 2022 May 31;12:891676. doi: 10.3389/fonc.2022.891676 (PMC9195174; doi:10.3389/fonc.2022.891676)
Supplement: Supplementary file 1 [file Table_1.docx]

**Table S1 Summary of different methods to construct personalized gene interaction networks.**

| Methods | Description | Software website | Input |
| --- | --- | --- | --- |
| SSN | Construct tumor sample specific network for each individual patient based on statistical perturbation analysis of tumor sample against a group of given control samples | https://github.com/xp-liu/SSN | Gene expression data of a single tumor sample and a group of given control samples |
| LIONESS | Reconstruct tumor sample specific network for each individual patient  in a population of tumor samples | https://github.com/WilfongGuo/Benchmark_control | Gene expression data of  a group of tumor samples |
| SPCC | Decompose each PCC measurement into multiple additive elements that form a new vector embedding correlation-like information of two variables for one tumor sample. | https://github.com/WilfongGuo/Benchmark_control | Gene expression data of  a group of tumor samples |
| CSN | Determine the gene–gene association by the statistical independency of two genes. | https://github.com/wys8c764/CSN | Gene expression data of  a group of tumor samples |
| Paired-SSN | The differential coexpression network between normal sample network and tumor sample network for each patient | https://github.com/NWPU-903PR/PNC | Gene expression data of a paired tumor/normal sample for a patient and a group of given control samples |
