# Supplementary material for: Network Control Models With Personalized Genomics Data for Understanding Tumor Heterogeneity in Cancer
Source: Front Oncol. 2022 May 31;12:891676. doi: 10.3389/fonc.2022.891676 (PMC9195174; doi:10.3389/fonc.2022.891676)
Supplement: Supplementary file 2 [file Table_2.docx]

**Table S2. Concept comparisons between different network control methods.**

| Methods | Network Styles | Dynamics | Targeted state | Input |
| --- | --- | --- | --- | --- |
| MMS | Directed networks | Local nonlinear | Any | Adjacency matrix |
| MDS | Undirected networks | Nonlinear | Any | Adjacency matrix |
| DFVS | Directed networks | Nonlinear | Attractors | Adjacency matrix |
| NCUA | Undirected networks | Nonlinear | Attractors | Adjacency matrix |
